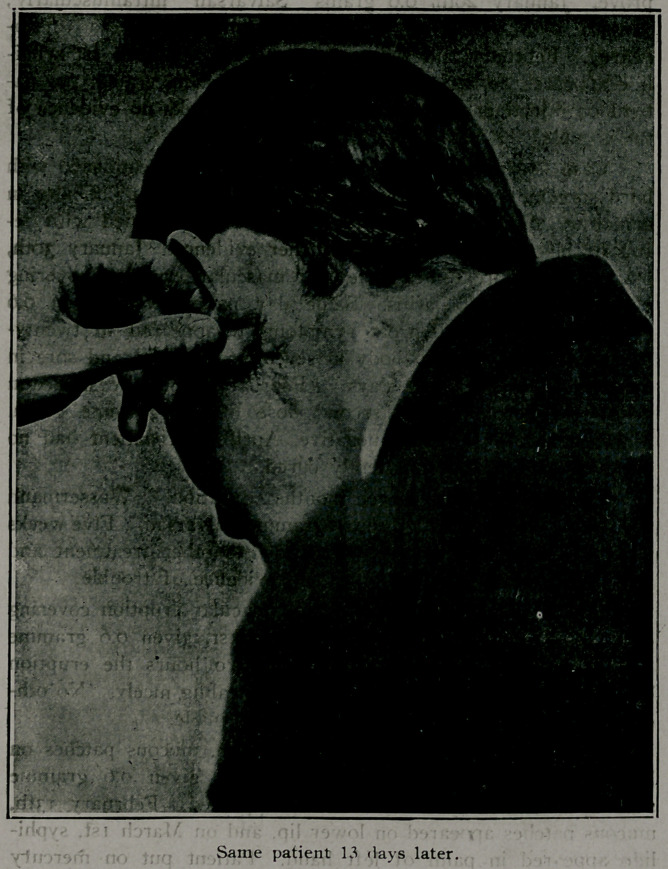# Salvarsan, or “606,” in the Treatment of Syphilis, with Report of Cases

**Published:** 1911-05

**Authors:** W. L. Champion

**Affiliations:** Atlanta, Ga.


### SALVARSAN, OR "606," IN THE TREATMENT OF SYPHILIS, WITH REPORT OF CASES.

By W. L. Champion, M.D., Atlanta, Ga.

There has not been in recent years a discovery made that has attracted more attention than Ehrlich's Salvarsan or "606" for the treatment of syphilis. The impression is prevalent that one dose will thoroughly eradicate the infection. This may be true in a limited number of cases, but it will take some time to know if this can be accomplished, and also to know the true value of the preparation and the best method of administering it. This impression has already done harm, and if not corrected by the physician who administers the new remedy, the patient leaves with the false hope of a cure to return later with more serious evidences of the dread malady.

That Ehrlich has discovered a remedy which is distinctly in advance of any heretofore used in the treatment of syphilis, there can be no doubt. A single injection is equal to several weeks' or months' treatment with mercury, and the patient avoids the unpleasant task of taking medicine daily. Its action is prompt in cases where mercury has failed, improvement in appearance and well-being is frequently marked.

Where immediate action is needed, we have in Salvarsan a valuable adjunct to our old and tried anti-syphilitic remedies.

The promptness with which it destroys the spirochete, and causes the healing of mucous membrane lesions certainly makes the syphilitic less dangerous to society from the standpoint of infection. While mercury is destructive to the spirochete, it cannot be as effectual as salvarsan, judging from the promptness with which lesions of the mucous membrane, skin and nervous system disappear.

That relapses will occur after a single dose there can be no doubt, as will be shown in the report of cases to follow. This fact will make it necessary to repeat the dose as each individual case may require, either from the return of symptoms, or a positive Wassermann reaction. I am using the preparation both intramuscularly and intravenously, when injected into the muscle it is slowly absorbed, and makes a hard mass that is sensitive to the touch for several days and sometimes two or three weeks.

The freedom from pain after the intravenous injection gives it the advantage over the intramuscular, and the fact that it is thrown direct into the circulation makes its action more powerful and certain.

It is stated by Weschellmann that the arsenic is eliminated more quickly when the preparation is administered intravenously —that is, in three days, while in the subcutaneous method it takes twelve to fourteen days. The fact that arsenic has been found in the glutens muscle in patients brought to autopsy several weeks after having had an injection may account for some failures, the drug not being absorbed sufficiently.

With the discovery of the spirochete, the Wassermann reaction and Ehrlich's Salvarsan, there has been awakened new interest in the management and treatment of syphilis, that is invaluable to the human race, we now have a more definite idea as to when or whether a patient is cured.

Tne newspaper and magazine advertisements the new remedy brought forth, have aroused the old syphilitic who has been taking his favorite prescription, or consulting the corner drug store man to go to his physician for an examination, to have his blood tested, to get a dose of "606." While the sensational articles that have appeared in the lay press claiming a one-dose cure for syphilis have been harmful, the knowledge the public has gained and the interest manifested will be helpful, as I believe the education of the public in regard to venereal diseases, their dangers, prevention and proper treatment is the only solution of the problem.

With each dose of salvarsan there are directions given— how to prepare the mixture and how to administer it, which, if followed, will make the preparation harmless if used in the proper cases. In administering the dose intravenously I use the median basilic vein at the middle third of the forearm, cocainize at the point of incision, expose the vein and make a slit large enough to admit a small canular. I use a canular in preference to a needle forced into the vein, so as to prevent the solution

Taken April 3rd, the day patient was given 0.6 gramme Salvarsan intramusculary.

from escaping into the surrounding tissue. But this can be avoided when the needle is used, by having two containers, one with normal salt solution and the other with the Salvarsan solution, using a three-way stop cock, so as first to allow a small quantity of the salt solution to flow into the vein to see that the needle is in proper position—then cut in the Salvarsan solution. It should require ten or fifteen minutes for the 250 or 300 cubic centimeters of the solution to flow into the vein.

For the intramuscular injection the solution or suspension should be neutral which will lessen the pain. I have not used a drop of acid in making the mixture in any case I have treated. Place the contents of an ampule in a glass mortar, add ten drops of caustic soda (15%) solution, rub well and then add five cubic centimeters of sterile water and mix thoroughly. The emulsion will be neutral nine times out of ten. If found acid, add another drop of soda solution. The mixture when taken into the syringe for injection measures five cubic centimeters. I use a record syringe with a large platinum needle that will carry the suspension. Clean the gluteal region with alcohol, paint with tincture of iodine and inject deep into the upper and outer portion of the muscle. I insert the needle first so as to see that a vessel is not punctured, then connect the syringe and press the piston slowly until the syringe is empty, that is to say, if the entire dose of 0.6 gramme is to be given. Massage gently over the point of injection so as to distribute the solution. Of the large number of cases injected I have not had a single abscess, nor has it been necessary to open up any case at the site of injection.

As advised, I administered the remedy to the first six cases in the hospital, seeing no elevation of temperature, very little pain, in fact, only three out of fifty-four were given morphia, so I gave the dose to the other patients in my office.

The fifty-four cases in this report are private patients that I have been able to keep in touch with since giving the remedy. As the report would be too lengthy to give all the cases in detail, only those of interest will be reported. Those cases that have shown a relapse I do not attribute to any fault in the technique of administering the drug, as it was given in the man-

Same patient 13 days later.

ner as those in which there was such a rapid disappearance of symptoms, and still free of any evidence of the disease.

Case 1—Papulo-squamous circinate patches on face, arms and legs. Patient had taken mercury two months, did not improve. January 26th, 0.6 grams Salvarsan intramuscularly; January 29th, patches fading, and on January 31st, had disappeared. February 2nd Wassermann negative; March 1st syphilide appeared on right ear; March 5th, patch ulcerated, put patient on injections of mercury and in two weeks no evidence of the trouble.

Case 2—January 23rd, patient came for treatment with hard sore in meatus almost closing the canal, great difficulty in urinating, Wassermann positive. Patient not satisfied with result of blood test, waited for further evidence. January 30th, marked sore throat, headache and mascular eruption covering abdomen, back and arms. Same day patient was given 0.6 gramme Salvarsan. Throat symptoms disappeared in twenty-four hours, eruption on body in seventy-two hours and sore in meatus healed in seven days. February 10th, Wassermann positive. February 11th, second dose of 0.6 gramme given. March 15th, Wassermann negative, April 15th, patient had no evidence of syphilis—apparently cured.

Case 3.—Syphilis eleven months duration. Wassermann positive. January 31st, given 0.6 gramme Salvarsan. Five weeks later Wassermann negative. Has had no other treatment and ten weeks since dose was given, no evidence of trouble.

Case 4.—Chancre in meatus and macular eruption covering entire body and extremities. January 31st, given 0.6 gramme Salvarsan intramuscularly. In seventy-two hours the eruption had practically disappeared and chancre healing nicely. No other evidence of syphilis, no Wassermann made.

Case 6.—Syphilis five months duration, mucous patches on lips and tonsils ulcerated. February 5th, given 0.6 gramme salvarsan. February 10th, ulcers disappeared. February 13th, mucous patches appeared on lower lip, and on March 1st, syphilide appeared in palm of left hand. Patient put on mercury and syphilide disappeared in three weeks. April 16th, patient hoarse, administered 0.6 gramme salvarsan intravenously and on April 19th voice was clear.

Case 7.—Contracted syphilis in July, 1909; mercury and iodide taken about nine months. February 9th, 1911, noticed left foot turned inward; had lost control of middle joint and toes, sensation lost in calf of leg, muscles flabby, and patient nervous and memory failing. Wassermann positive. February 9th, administered 0.6 gramme salvarsan intramuscularly. February 13th, patient feeling better, regaining control of leg and foot and sensation returning. February 21st, had gained five pounds, foot and leg almost normal. March 9th, perfect use of leg and foot. Sensation normal and patient had gained fourteen pounds. Wassermann negative.

Case 13.—Syphilis two months duration, both tonsils badly ulcerated, patches around arms and general grandular enlargement. Had been taking patent medicine called B. B. B. February 15th gave 0.6 gramme Salvarsan intramuscularly. February 15th gave 0.6 gramme Salvarsan intramuscularly. February 17th, tonsils clean and healing and throat symptoms better, patches around arm not improved. March 3rd, throat symptoms returned. March 4th, second dose of 0.6 gramme Salvarsan. March 7th, patient improving. March 15th, patches around arms healed. March 20th, tonsil ulcerated, put patient on mercury.

Case 15.—Student, syphilis six months duration. Had been given dose of Salvarsan by another physician three weeks before consulting me. The first dose cured syphilide of the palm, but did not improve the laryngitis, which was very severe, nor patch on tongue. On February 16th, gave patient 0.6 gramme of Salvarsan. February 20th, voice clearing up and on 26th normal. On April 1st, two mucous patches appeared on lower lip. April 9th gave 0.6 gramme Salvarsan intravenously. April 13th, patches had disappeared and voice normal.

Case 17.—Syphilis four months duration. Syphilis of tongue which had persisted in spite of mercury, potash, cacodylate of soda, and a visit to Hot Springs. February 18th, 0.6 gramme Salvarsan. April 15th, no improvement.

Case 20.—Upper and lower lips covered with mucous patches and sides of tongue ulcerated. February 19th, full dose of Salvarsan. March 3rd, lips and tongue normal.

Case 26.—Intense papular eruption covering both arms and legs, had been present two weeks. Patient had been treated several months before with mercury and potash. March 2nd, gave full dose of Salvarsan intramuscularly; March 16th, very little of eruption left, and March 27th skin clear.

Case 33.—Syphilis fourteen months duration. Papulo-squamous patches on arms and legs. March 19th gave 0.5 gramme Salvarsan intravenously, March 25th patches gone.

Case 35.—Physician, chancre on thumb in December, 1910. Took mercury badly. March 21st, laryngitis, very hoarse, throat ulcerated, pains in elbows and knees. Patient very nervous and had not slept in several nights. March 21st gave 0.6 gramme Salvarsan intramuscularly. In twenty-four hours voice clearing rapidly and in forty-eight hours practically normal. The pains in throat and joints were so quickly relieved that patient slept well the first night after the injection. April 15th, no return of symptoms.

Case 36—Female, infected with syphilis in November, 1910. Had been given mercury and potash. Mucous patches in mouth and scaling syphilides of palms and soles. March 21st 0.5 gramme salvarsan, March 27th, mouth normal and syphilides on hands and feet disappearing. April 7th, patient apparently well.

Case 41.—Contracted syphilis in 1906, Wassermann positive, deep tubercular syphilide behind left ear and in neck, right elbow three inches larger than left. Patient was in constant pain and had slept very little in several weeks. The syphilides had been on neck and ear for three months. April 3rd, 0.6 gramme Salvarsan intramuscularly. In forty-eight hours the patient was free of pain, and in seventy-two hours the sores were cleaned off and healing. Elbow that was swollen reduced one inch. On April 17th, two weeks after injection the sores were almost healed, as the accompanying pictures will show.

At is has been only a few weeks since the drug has been administered to a number of the cases the Wassermann test has not been made.

One case of tabes with negative Wassermann, no improvement was noted. In another with a positive Wassermann, the pains were lessened, the patient gained in weight and was able to indulge his sexual appetite.

Of the fifty-four cases treated, ten had no lesions of the mucous membrane or skin where the effect of the drug could be watched, but these ten gave positive Wassermann or had evidence of the disease a few weeks before. Forty-six had lesions at the time the drug was given, and all visible lesions healed in forty-three cases, two improved, but did not heal entirely. Six cases out of the fifty-four relapsed and were either given the second dose or put on mercury.

## Figures and Tables

**Figure f1:**
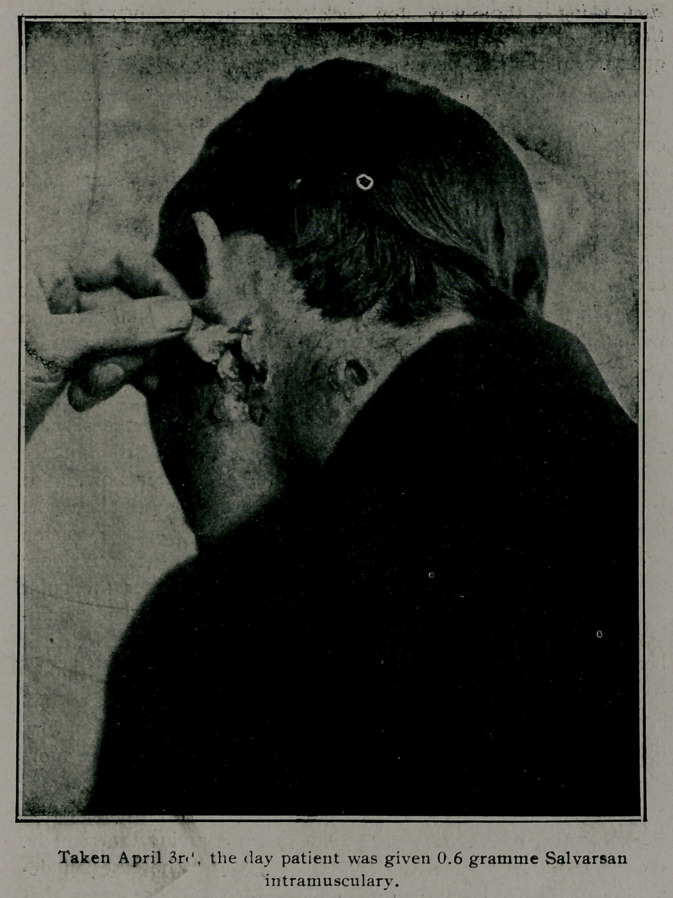


**Figure f2:**